# Social determinants associated with psychological distress in children and adolescents during and after the first COVID-19-related lockdown in France: results from the CONFEADO study

**DOI:** 10.1186/s12889-023-16284-5

**Published:** 2023-07-18

**Authors:** Mégane Estevez, Nicolas Oppenchaim, Dalila Rezzoug, Isaura Laurent, Sandrine Domecq, Imane Khireddine-Medouni, Xavier Thierry, Cédric Galera, Carla De Stefano, Stéphanie Vandentorren

**Affiliations:** 1 Centre de recherche Bordeaux Population Health (BPH), Université de Bordeaux, Inserm U1219, Bordeaux, France; 2 Université de Tours, UMR CITERES 7324, 35 Allée Ferdinand de Lesseps, Tours, 37200 France; 3 Université Paris 13 Sorbonne Paris Nord, Bobigny, France; 4 AP-HP, Department of Child and Adolescent Psychiatry and General Psychiatry, Avicenne Hospital, Bobigny, France; 5 Centre de Recherche en épidémiologie et santé des populations (CESP), Université Paris Saclay, Villejuif, France; 6 AP-HP, Urgences - Samu 93, Hôpital Avicenne, Bobigny, France; 7 École nationale de la statistique et de l’analyse de l’information (ENSAI), Bruz, France; 8 Santé publique France, Saint Maurice, France; 9 ELFE Joint Unit, French Institute for Demographic Studies (Ined), French Institute for Medical Research and Health (Inserm), Paris, France; 10 Department of Child and Adolescent Psychiatry, Centre Hospitalier Charles Perrens, Bordeaux, France; 11 Centre National de Ressources et de Résilience Lille-Paris (CN2R), Lille, France

**Keywords:** COVID-19, Lockdown, Children, Adolescents, Living and housing conditions, Social determinants, Psychological distress

## Abstract

**Background:**

This study aimed to analyze the parental socio-demographic characteristics of children and adolescents aged 9 to 18 years old, as well as the living and housing conditions associated with the psychological distress in these two sub-populations during and after France’s first national COVID-19-related lockdown in spring 2020.

**Methods:**

We used data from the cross-sectional, observational, web-based study CONFEADO, which collected data on children and adolescents’ living and housing conditions and socio-demographic characteristics as well as those of their parents. It also collected data on children’s and adolescents’ health behaviors and psychological distress. We assessed psychological distress using the 10-item Children and Adolescents Psychological Distress Scale (CAPDS-10), and performed a multinomial logistic regression.

**Results:**

A total of 2882 children and adolescents were included in the present study. Factors associated with moderate psychological distress included being a female, parental financial difficulties, a lack of a private living space at home for the child/adolescent, and the following child health behaviors: no leisure or recreational activities with adults in the household, doing less than one hour of school homework a day, and not going outside during the lockdown. Severe psychological distress was associated with the parent’s occupation (especially essential frontline workers), a lack of a private living space at home for the child/adolescent, and the following child health behaviors: spending over 5 h a day on social media, doing less than one hour of school homework a day, and no leisure or recreational activities with adults in the household.

**Conclusions:**

This study emphasizes the impact of housing and living conditions, as well as parents’ socio-economic characteristics on children’s health behaviors and psychological needs during the first COVID-19-related lockdown in France. Our results suggest that health policies implemented during future pandemics should consider these structural social determinants to prevent severe psychological distress in children and adolescents.

## Background

In order to limit the spread of SARS-CoV-2 during the two years of the COVID-19 pandemic, most affected countries introduced unprecedented radical preventive measures, including lockdowns. This increased the burden of mental health in general populations, especially more isolated people, as well as those with difficult economic, housing, and family structure (e.g., single parents) conditions [1]. National strategies were also implemented to cope with the huge socioeconomic consequences of the pandemic (shorter working hours, increased unemployment benefit, etc.). In France, the government allowed shorter working hours at no cost to employers and implemented national lockdowns; the first lasted 8 weeks from 17 March to 11 May 2020. Schools were closed, with classes and homework continuing remotely. There were restrictions on family visits and local and international travel [2]. People had to remain at home; they were allowed outside for one hour a day provided they followed social distancing rules. Most socialization and care structures were closed [3].

This pandemic was a challenging period for children and adolescents (grouped under the single term ‘children’ where appropriate hereafter) worldwide, especially as late childhood and adolescence are critical periods for development and mental well-being [4]. School closures increased young people’s vulnerability to mental health problems, through reduced social interaction and physical activity, as well as increased screen time, irregular sleep patterns, and sub-optimal nutrition (especially in children for whom free school meals were an important source of nutrition [5], and in countries like France where the cost of fee-based school lunches was determined by family income [6]). These factors also negatively impacted children’s well-being in the short and long terms [7–11]. Younger people had few resources and few prior life experiences to be able to deal with stressful situations like COVID-19 [12]. A systematic review of the psychological impact of related lockdowns on children identified three studies reporting depression prevalence rates between 22.6% and 43.7%, and two studies reporting anxiety symptom rates between 18.9% and 37.4% [13].

Children from vulnerable social backgrounds, especially girls, were most affected by the impact of COVID-19 [14]. Parenting during the pandemic was an additional challenge for single families, for those on a low income, and those living in crowded housing. These families had to deal with remote learning (children), remote working (parents), and a greater number of household chores [15]. Previous studies on the risk factors associated with poorer mental health among children and adolescents during the pandemic highlighted several predictors as follows: social isolation, screen time and excessive social media use, parental stress, a poor parent-child relationship, low socioeconomic status, preexisting mental health conditions, and/or preexisting disabilities [16]. However, in France, very few such studies have been conducted [17, 18], and none directly interviewed children or examined household-related structural social determinants (e.g., parent’s economic conditions and family structure) that could have affected their behavior and mental health during and after the pandemic.

We hypothesize that the living and housing conditions of many families, as well as the socio-demographic characteristics of parents during COVID-19-related lockdowns influenced children’s health behaviors, and had a considerable impact on the mental health of this population. Accordingly, we aimed to identify at-risk and protective structural social determinants associated with psychological distress in children and adolescents aged 9 to 18 years during and after France’s first COVID-19-related lockdown in spring 2020. The results from this study could inform both the development of interventions aimed at improving the mental health of children and adolescents’ during future pandemic-related lockdowns, and actions to address the issue of widening social inequity.

## Methods

### Design and study population

CONFEADO (*CONFinement, Enfant et ADO*, translated as Lockdown, Child and Adolescent) is a cross-sectional observational study that examined how children experienced the first COVID-19-related lockdown in France. It was conducted between 9 June and 14 September 2020. Children from CONFEADO who were in the care of child protection services were not included in the present analyses because data (parent-provided) on economic, living and housing conditions were not collected for this sub-population.

One of the child’s parents or his/her legal guardian had to provide consent for the child to participate. They also completed the parent questionnaire. Furthermore, children themselves had to provide their consent. In the present study, only children aged 9 to 18 years old who completed the 10-item Children and Adolescent’s Psychological Distress Scale (CAPDS-10) for the assessment of psychological distress were included.

### Data and procedure

CONFEADO used a web-based anonymous, standardized questionnaire, which was completed by one of the participating child’s parents (first part) and by the child him/herself (second part). The link to the questionnaire was sent to families by various institutions, associations, and partners of *University Sorbonne Paris Nord*, *Santé publique France* (SpF), *Observatoire national de la protection de l’enfance* (ONPE), *Assistance Publique-Hôpitaux de Paris* (AP-HP). It was also distributed on social networks.

### Study variables

#### Children’s psychological distress

The CAPDS-10 questionnaire was specifically designed for the CONFEADO study and was validated in a previous study using data from CONFEADO [19]. It is a self-assessment scale based on internationally validated scales including the Child Behavior Checklist (CBCL) and the Youth Self Report (YSR), and is intended to provide a measure of child psychological distress at a given time. All ten items concern a respondent’s feelings over the previous two weeks. The first three assess his/her anxiety (nervousness/worry, stress, restlessness), the next three depression (lack of desire/pleasure in doing things, discouragement, lack of energy), the seventh somatic complaints (pain/fatigue/difficulty sleeping), and the remaining three aggressive behavior (opposition/irritability/disputes). Each item has four possible responses as follows: ‘never’, ‘sometimes’, ‘more than half of the time’, or ‘almost every day’, corresponding to a score between 0 (never) and 3 (almost every day). Accordingly, the total scale score ranges from 0 to 30; the higher the score, the more severe is the psychological distress. In the present study, the total score was analyzed as a three-modality categorical variable: scores between 0 and 9 indicated no or mild distress, scores between 10 and 18 indicated moderate distress, and scores between 19 and 30 indicated severe distress.

#### Socio-demographic characteristics of children and their parents

CONFEADO collected data on two socio-demographic characteristics of the child’s parents as follows: nationality (two French parents, one non-French parent, two non-French parents), and the level of education of the parent who completed the questionnaire (No diploma/Vocational training certificate, Upper secondary school certificate, Bachelor’s degree, Master’s degree/PhD/other).

For the present study, we constructed a categorical variable called ‘parent’s occupation’ using data collected from an open-ended question about this theme in CONFEADO which parents answered. In order to do this, we first created occupation clusters to group together occupations in the education sector (teachers, professors, etc.), healthcare professionals (doctors, nurses, psychologists, pharmacists, midwives, psychotherapists, etc.), essential frontline workers (housekeepers, mail carriers, drivers, cashiers, bakers, truck drivers, police officers, etc.), care providers (childcare providers, caregivers, nursing assistants, home aides, etc.), and commercial actors (florists, hairdressers, etc.). Occupations that did not fit into any of these six clusters and whose socio-professional category was known were added to one of the following three categories: ‘other executive occupations’, ‘other intermediate occupations’, ‘other employee/manual worker occupations’.

The categorical variable ‘parent’s occupation’ was then constructed as follows: ‘frontline’ and ‘care providers’ were grouped together into one modality (their commonality being that they continued to work after the lockdown started); ‘healthcare professionals’ and ‘other occupations’ comprised two extra modalities, with ‘education sector’ and ‘commercial actors’ included in the latter. Finally, there was an ‘unknown’ modality for those whose occupation was unknown.

CONFEADO collected data on two socio-demographic characteristics of children: sex and age. Both were provided by the child (i.e., not by the parent) in the second part of the questionnaire (see above). Age was categorized into three age classes (9–11, 12–14, and 15–18 years) which correspond to different developmental stages of childhood and adolescence.

#### Living conditions

The two following variables were used: parent’s self-perceived financial situation (very comfortable, comfortable, getting by, difficult to make ends meet) and family structure (two-parent, stepparent, single-parent). Both were provided by the parent (first part of questionnaire, see above).

#### Housing conditions

The three following variables were used: (i) municipal population density, categorized into ‘highly’, ‘moderately’, ‘sparsely’, and ‘very sparsely’ populated area; this variable was constructed from data on the child’s place of residence, using the municipal density grid developed by the National Institute of Statistics and Economic Studies’s (INSEE; *Institut National de la Statistique et des Études Économiques*), which classifies districts in France into 4 categories based on municipal population density; (ii) type of housing (house with garden, house with no garden/apartment/other); (iii) a private space in the accommodation if the child needed it (yes, no). The latter variable was provided by the child. The other two were provided by the parent.

#### Child mental health-related behaviors

Variables on the following child mental health-related behaviors during and after the lockdown were collected: how frequently the child went outside during the lockdown (never, approximately once a week, approximately 3 times a week, every day, once a day, multiple times a day), the time spent every day on social media since the beginning of the lockdown (none, less than an hour, 1 to 3 hours, 3 to 5 hours, more than 5 hours), the frequency of leisure and recreational activities with adults in the household since the beginning of the lockdown (including board games, video games, sports, and manual activities such as gardening, cooking, sewing, etc.) (never, approximately once a week, approximately 3 times a week, every day), and the time spent on school homework every day since the beginning of the lockdown (less than one hour, one hour, 2 to 3 hours, more than 3 hours). All these variables were provided by the children.

#### Child mental health and other variables

The following two variables were collected in CONFEADO and analyzed in the present study as they can be considered confounding factors in the relationship between social factors and psychological distress: history of child mental health disorder (yes, no) and child experience of a painful and/or stressful event (e.g., parents’ divorce, death of a loved one, or a romantic disappointment) in the previous 12 months (yes, no). They were provided by the parent and the child, respectively. Data on COVID-19-related infections and hospitalizations of a loved one were also collected (provided by the child).

### Statistical analysis

**Fig. 1:**
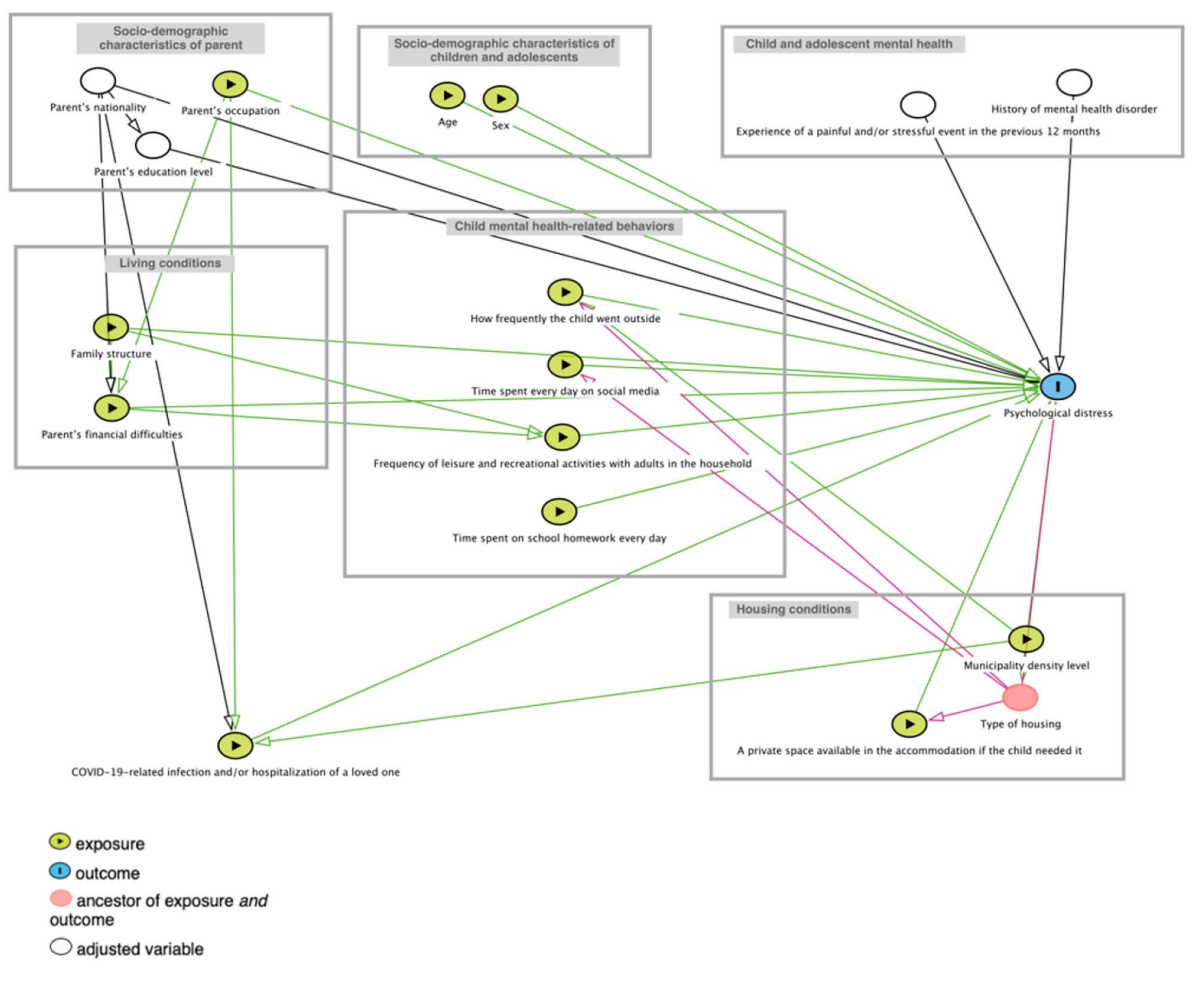
Directed acyclic graph (DAG) representing causal relationships between explanatory variables and child and adolescent psychological distress, CONFEADO study, France, 2020

We developed a DAG (directed acyclic graph) (Fig. 1) to represent the relationships between the explanatory variables of interest, known confounding factors, and child psychological distress, using an etiological explanatory approach. The DAG allowed us to select a subset of relevant variables to include in our final model, according to the conceptual framework of the social determinants of COVID-19 on children’s health and wellbeing [14].^1^

We first conducted univariable analyses for each explanatory factor to explore the relationship between each independent variable and the dependent variable, ignoring the potential effect of other explanatory variables. Then, we performed a multivariable multinomial logistic regression model on the complete dataset to investigate all explanatory variables, including the social factors associated with psychological distress. We were interested in the associations between parental socio-demographic characteristics (parent’s occupation), living and housing conditions (variables that usually impact psychological distress, and may have influenced child health behaviors during the lockdown), age, sex, and whether a relative had been infected with COVID-19 and/or hospitalized for it. We adjusted for the parent’s education level, nationality, child history of mental disorders, whether the child had experienced a painful and/or stressful event in the previous 12 months, and the type of housing. Application conditions were verified before running the model, including collinearity between variables and sufficient sample size. After running the model, fit and performance were evaluated. All analyses were performed using R version 4.2. The significance level was set at 5%.

## Results

### Sample characteristics

**Fig. 2:**
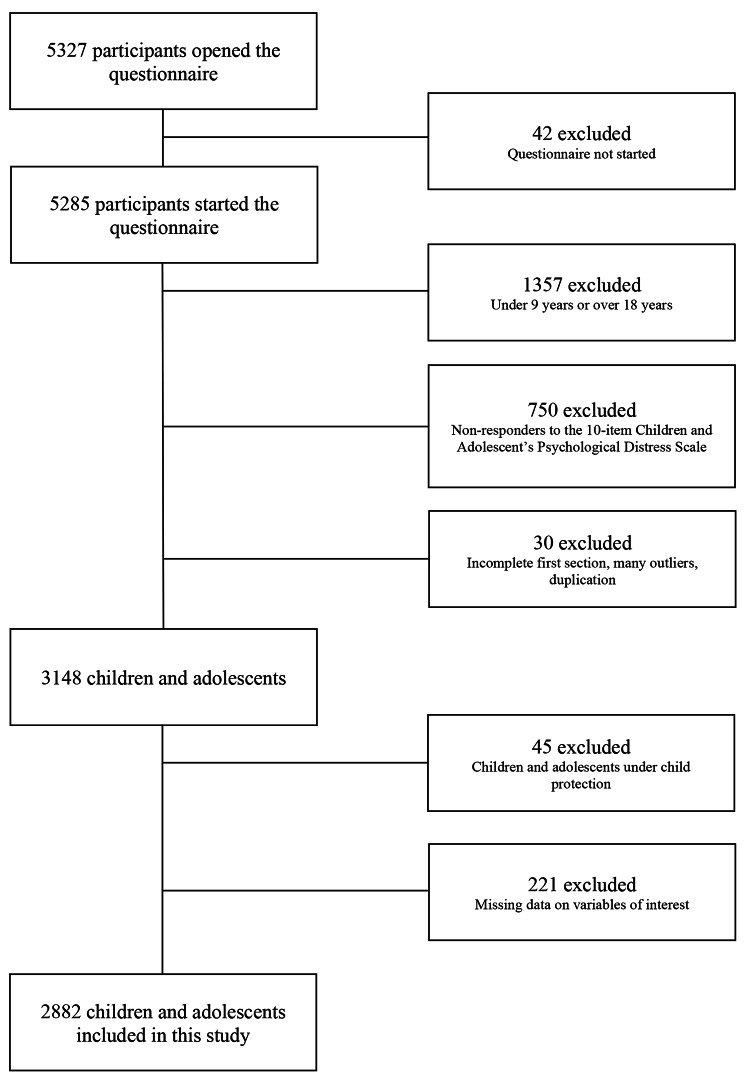
Flow chart of study sample, CONFEADO study, France, 2020

The present study included a total of 2882 children (Fig. 2; Table 1). Of these, 2005 (69.6%) were females, 393 (13.6%) were between 9 and 11 years old, 469 (16.3%) between 12 and 14 years old, and 2020 (70.1%) between 15 and 18 years old. Concerning child mental health, 887 (30.8%) and 201 (7%) had moderate and severe psychological distress, respectively, while 639 (22.2%) had a history of mental disorders; 1228 (42.6%) had had a painful and/or stressful event in the previous 12 months. In terms of child health behaviors, 536 (18.6%) went outside multiple times a day during the lockdown, 594 (20.6%) had spent more than 5 hours a day on social media since the lockdown began, and 643 (22.3%) had participated in leisure and recreational activities with adults in their household every day since the lockdown started. With regard to children’s housing conditions, 438 (15.3%) did not have a private space in their living accommodation when needed. In terms of school work at home, 826 (28.7%) had spent more than 3 hours a day on school homework since the lockdown started. With regard to the impact of the COVID-19 pandemic on relatives, 910 (31.6%) had had a relative infected and/or hospitalized due to COVID-19.

**Table 1:** Characteristics of child and adolescent study participants, CONFEADO study, France, 2020. (N = 2882)

Variable	N (%)
**Socio-demographic characteristics of children and adolescents**	
*Sex*	**2882**
Male	877 (30.4)
Female	2005 (69.6)
*Age (years)*	**2882**
9–11	393 (13.6)
12–14	469 (16.3)
15–18	2020 (70.1)
**Child and adolescent mental health**	
*Psychological distress*	**2882**
No or mild distress	1794 (62.2)
Moderate distress	887 (30.8)
Severe distress	201 (7.0)
*History of mental health disorder*	**2882**
No	2243 (77.8)
Yes	639 (22.2)
*Experience of a painful and/or stressful event in the previous 12 months*	**2882**
No	1654 (57.4)
Yes	1228 (42.6)
**Child mental health-related behaviors**	
*How frequently the child went outside during the first French lockdown*	**2882**
Never	443 (15.4)
Approximately once a week	757 (26.3)
Approximately 3 times a week	514 (17.8)
Every day, once a day	632 (21.9)
Multiple times a day	536 (18.6)
*Time spent every day on social media since the beginning of the first French lockdown*	**2882**
None	394 (13.7)
Less than an hour	332 (11.5)
1 to 3 h	826 (28.7)
3 to 5 h	736 (25.5)
More than 5 h	594 (20.6)
*Frequency of leisure and recreational activities with adults in the household since the beginning of the first French lockdown*	**2882**
Never	648 (22.5)
Approximately once a week	862 (29.9)
Approximately 3 times a week	729 (25.3)
Every day	643 (22.3)
*Time spent on school homework every day since the beginning of the first French lockdown*	**2882**
Less than one hour	527 (18.3)
One hour	536 (18.6)
2 to 3 h	993 (34.5)
More than 3 h	826 (28.7)
**Housing conditions**	
*A private space available in the accommodation if the child needed it*	**2882**
No	438 (15.3)
Yes	2444 (84.8)
**Impact of the COVID-19 pandemic on relatives**	
*COVID-19-related infection and/or hospitalization of a loved one*	**2882**
No	1972 (68.4)
Yes	910 (31.6)

In terms of parents’ socio-demographic characteristics (Table 2), 211 (7.3%) were not French nationals. Concerning the respondent parent, 696 (24.1%) had no educational diploma, 348 (12.1%) were essential frontline workers, and 252 (8.7%) were healthcare professionals. With regard to living conditions, 686 (23.8%) were single-parent families, and 319 (11.1%) found it difficult to make ends meet. With regard to housing conditions, 679 (23.6%) lived in a very sparsely populated area, and 881 (30.6%) lived in a house without a garden or an apartment.

**Table 2:** Characteristics of the parents of children and adolescents included in the study sample, CONFEADO study, France, 2020. (N = 2882)

Variable	N (%)
**Socio-demographic characteristics of parent**	
*Parent’s nationality*	**2882**
Two French parents	2301 (79.8)
One non-French parent	370 (12.8)
Two non-French parents	211 (7.3)
*Parent’s level of education*	**2882**
No diploma/Vocational training certificate	696 (24.1)
Upper secondary school certificate	636 (22.1)
Bachelor’s degree	735 (25.5)
Master’s degree, PhD, other	815 (28.3)
*Parent’s occupation*	**2882**
Essential frontline worker	348 (12.1)
Healthcare professional	252 (8.7)
Other occupation	1681 (58.3)
Inactive	424 (14.7)
Unknown	177 (6.1)
**Living conditions**	
*Family structure*	**2882**
Two-parent	1861 (64.6)
Stepparent	335 (11.6)
Single-parent	686 (23.8)
*Parent’s financial difficulties*	**2882**
Very comfortable	524 (18.2)
Comfortable	1100 (38.2)
Getting by	939 (32.6)
Difficult to make ends meet	319 (11.1)
**Housing conditions**	
*Municipal population density*	**2882**
High	1086 (37.7)
Moderate	754 (26.2)
Sparse	363 (12.6)
Very sparse	679 (23.6)
*Type of housing*	**2882**
House with garden	2001 (69.4)
House with no garden, apartment, other	881 (30.6)

### Factors associated with psychological distress in univariable analysis

The univariable results revealed significant associations between moderate and/or severe psychological distress and several explanatory factors (Table 3) such as sex, age, two French parents, parent’s level of education, parental inactivity, single-parent family status, parental financial difficulties, type of housing, availability of private space within the living accommodation, history of mental health disorders, experiencing painful and/or stressful events in the previous 12 months, never going outside during the lockdown, spending more than one hour per day on social media since the beginning of the lockdown, never engaging in leisure or recreational activities with adults in the household since the beginning of the lockdown, and spending less than one hour per day on school homework since the beginning of the lockdown. Additionally, having a loved one infected or hospitalized due to COVID-19 was also associated with moderate and severe psychological distress.

**Table 3:** Factors associated with psychological distress in children and adolescents (9–18 years old) during and after the first COVID-19 pandemic-related lockdown in France in spring 2020: univariable multinomial logistic regression, CONFEADO study, France, 2020. (N = 3103)

Variable	Moderate distress vs. No or mild distress		Severe distress vs. No or mild distress	
	OR [95% CI]	p	OR [95% CI]	p
**Children and adolescents socio-demographic characteristics**				
*Sex*				
Male (Ref)	-		-	
Female	**2.58 [2.14; 3.11]**	**< 0.001**	**2.48 [1.77; 3.47]**	**< 0.001**
*Age (years)*				
9–11 (Ref)	-		-	
12–14	0.97 [0.70; 1.34]	0.853	1.89 [0.94; 3.81]	0.075
15–18	**2.24 [1.74; 2.89]**	**< 0.001**	**4.19 [2.31; 7.61]**	**< 0.001**
**Parent’s socio-demographic characteristics**				
*Parent’s nationality*				
Two French parents	0.80 [0.63; 1.00]	0.052	**0.50 [0.35; 0.72]**	**< 0.001**
One non-French parent (Ref)	-		-	
Two non-French parents	1.36 [0.96; 1.94]	0.088	1.37 [0.82; 2.29]	
*Parent’s level of education*				
No diploma/Vocational training certificate (Ref)	-		-	
Upper secondary school certificate	**0.75 [0.60; 0.94]**	**0.013**	**0.68 [0.47; 1.00]**	**0.049**
Bachelor’s degree	**0.68 [0.55; 0.85]**	**< 0.001**	**0.55 [0.38; 0.81]**	**0.002**
Master’s degree, PhD, other	**0.72 [0.58; 0.89]**	**0.002**	**0.61 [0.420; 0.87]**	**0.007**
*Parent’s occupation*				
Essential frontline worker	1.17 [0.92; 1.50]	0.208	**1.98 [1.35; 2.91]**	**< 0.001**
Healthcare professional	0.83 [0.63; 1.11]	0.221	0.94[0.55; 1.59]	0.805
Inactive	**1.29[1.03; 1.60]**	**0.025**	**1.82 [1.27; 2.62]**	**0.001**
Unknown	1.01 [0.73; 1.41]	0.942	1.25 [0.71; 2.22]	0.436
Other occupation (Ref)	-		-	
**Living conditions**				
*Family structure*				
Two-parent (Ref)	-		-	
Stepparent	1.06 [0.83; 1.37]	0.630	1.30 [0.84; 2.01]	0.239
Single-parent	**1.23 [1.02; 1.48]**	**0.032**	**1.43 [1.03; 1.99]**	**0.032**
*Parent’s financial difficulties*				
Very comfortable (Ref)	-		-	
Comfortable	1.14 [0.90; 1.44]	0.270	1.59 [0.98; 2.58]	0.060
Getting by	**1.83 [1.45; 2.30]**	**< 0.001**	**2.90 [1.81; 4.65]**	**< 0.001**
Difficult to make ends meet	**1.83 [1.36; 2.46]**	**< 0.001**	**3.88 [2.27; 6.63]**	**< 0.001**
**Housing conditions**				
*Municipal population density*				
High	1.15 [0.95; 1.40]	0.157	1.28 [0.91; 1.81]	0.157
Moderate (Ref)	-		-	
Sparse	0.91 [0.69; 1.18]	0.473	0.59 [0.34; 1.04]	0.069
Very sparse	0.91 [0.73; 1.13]	0.395	1.16 [0.79; 1.70]	0.441
*Type of housing*				
House with garden	**0.69 [0.58; 0.81]**	**< 0.001**	**0.59 [0.45; 0.79]**	**< 0.001**
House with no garden, apartment, other (Ref)	-		-	
*A private space available in the accommodation if the child needed it*				
No (Ref)	-		-	
Yes	**0.35 [0.29; 0.44]**	**< 0.001**	**0.16 [0.12; 0.21]**	**< 0.001**
**Child and adolescent mental health**				
*History of mental health disorder*				
No (Ref)	**-**		**-**	
Yes	**4.83 [3.9737; 5.87]**	**< 0.001**	**14.24 [10.52; 19.26]**	**< 0.001**
*Experience of a painful and/or stressful event in the previous 12 months*				
No (Ref)	**-**		**-**	
Yes	**3.59 [3.05; 4.22]**	**< 0.001**	**8.55 [6.16; 11.86]**	**< 0.001**
**Child mental health-related behaviors**				
*How frequently the child went outside during the lockdown*				
Never	**2.98 [2.24; 3.97]**	**< 0.001**	**5.05 [2.99; 8.53]**	**< 0.001**
Approximately once a week	**2.49 [1.93; 3.22]**	**< 0.001**	**3.46 [2.09; 5.72]**	**< 0.001**
Approximately 3 times a week	**2.02 [1.53; 2.67]**	**< 0.001**	1.72 [0.96; 3.09]	0.068
Every day, once a day	**1.79 [1.36; 2.34]**	**< 0.001**	**2.13 [1.25; 3.64]**	**0.006**
Multiple times a day (Ref)	-		-	
*Time spent every day on social media since the beginning of the lockdown*				
None (Ref)	-		-	
Less than an hour	1.04 [0.73; 1.47]	0.826	**2.94 [1.33; 6.50]**	**0.008**
1 to 3 h	**1.54 [1.17; 2.04]**	**0.002**	**3.44 [1.68; 7.04]**	**< 0.001**
3 to 5 h	**2.24 [1.69; 2.96]**	**< 0.001**	**4.91 [2.41 10.03]**	**< 0.001**
More than 5 h	**3.24 [2.43; 4.33]**	**< 0.001**	**9.85 [4.86; 19.96]**	**< 0.001**
*Frequency of leisure and recreational activities with adults in the household since the beginning of the lockdown*				
Never	**2.24[1.76; 2.84]**	**< 0.001**	**4.62 [3.06; 6.99]**	**< 0.001**
Approximately once a week	**1.55 [1.23; 1.94]**	**< 0.001**	**1.77 [1.15; 2.73]**	**0.010**
Approximately 3 times a week	**1.29 [1.02; 1.63]**	**0.032**	0.70 [0.41; 1.20]	0.199
Every day (Ref)	-		-	
*Time spent on school homework every day since the beginning of the lockdown*				
Less than one hour	**1.75 [1.39; 2.21]**	**< 0.001**	**3.02 [2.10 4.35]**	**< 0.001**
One hour	1.08 [0.86; 1.36]	0.510	1.01 [0.66; 1.56]	0.960
2 to 3 h	1.01 [0.82; 1.23]	0.950	0.78 [0.53; 1.15]	0.210
More than 3 h (Ref)	-		-	
**Impact of the COVID-19 pandemic on relatives**				
*COVID-19-related infection and/or hospitalization of a loved one*				
No (Ref)	-		-	
Yes	**1.30 [1.10; 1.54]**	**0.002**	**1.59 [1.20; 2.10]**	**0.001**

OR: Odds Ratios; 95% CI: 95% Confidence Interval; p: p-value; Ref: Reference

### Factors remained associated with psychological distress in multivariable analysis

#### Risk factors for moderate psychological distress

The risk factors most strongly associated with moderate psychological distress (*versus* low or no psychological distress, i.e., the reference category) in the participating children are presented in Table 4. Multinomial logistic regression highlighted the following factors: being a female (aOR, 1.96 ; 95% CI, 1.57 ; 2.46), parental financial difficulties (aOR, 1.38 ; 95% CI, 1.04 ; 1.83), never going outside during the lockdown (aOR, 1.65 ; 95% CI, 1.15 ; 2.37), only going outside once a week (aOR, 1.48 ; 95% CI, 1.08 ; 2.03), or every day, once a day (aOR, 1.42 ; 95% CI, 1.05 ; 1.92), never doing leisure or recreational activities with adults in the household since the lockdown started (aOR, 1.53 ; 95% CI, 1.14 ; 2.04), and doing less than one hour a day of school homework since the beginning of the lockdown (aOR, 1.42 ; 95% CI, 1.08 ; 1.88).

**Table 4:** Factors associated with psychological distress in children and adolescents (9–18 years old) during and after the first COVID-19 pandemic-related lockdown in France in spring 2020: multinomial logistic regression, CONFEADO study, France, 2020. (N = 2882)

Variable	Moderate distress (N = 887) vs. No or mild distress (N = 1794)		Severe distress (N = 201) vs. No or mild distress (N = 1794)	
	aOR [95% CI]	p	aOR [95% CI]	p
**Children and adolescents socio-demographic characteristics**				
*Sex*				
Male (Ref)	-		-	
**Female**	**1.96 [1.57; 2.46]**	**< 0.001**	1.52 [0.98; 2.37]	0.062
*Age (years)*				
9–11 (Ref)	-		-	
12–14	0.78 [0.53; 1.15]	0.205	1.19 [0.50; 2.80]	0.695
15–18	1.13 [0.79; 1.63]	0.505	0.90 [0.40; 2.01]	0.793
**Parent’s socio-demographic characteristics**				
*Parent’s occupation*				
**Essential frontline worker**	0.94 [0.69; 1.27]	0.693	**1.66 [1.00; 2.75]**	**0.049**
Healthcare professional	0.93 [0.66; 1.31]	0.691	0.96 [0.49; 1.87]	0.901
Inactive	0.91 [0.69; 1.21]	0.526	1.11 [0.67; 1.84]	0.677
Unknown	0.81 [0.55; 1.18]	0.272	1.25 [0.63; 2.48]	0.526
Other occupation (Ref)	-		-	
**Living conditions**				
*Family structure*				
Two-parent (Ref)	-		-	
Stepparent	0.75 [0.56; 1.00]	0.072	0.85 [0.51; 1.42]	0.536
Single-parent	0.81 [0.64; 1.02]	0.072	0.67 [0.44; 1.01]	0.056
*Parent’s financial difficulties*				
Very comfortable (Ref)	-		-	
Comfortable	1.02 [0.78; 1.34]	0.882	1.23 [0.70; 2.16]	0.461
**Getting by**	**1.38 [1.04; 1.83]**	**0.025**	1.51 [0.86; 2.68]	0.153
Difficult to make ends meet	1.03 [0.70; 1.50]	0.896	1.08 [0.53; 2.18]	0.832
**Housing conditions**				
*Municipal population density*				
High	0.97 [0.76; 1.23]	0.785	1.01 [0.64; 1.58]	0.980
Moderate (Ref)	-		-	
Sparse	1.00 [0.74; 1.36]	0.997	0.86 [0.44; 1.66]	0.648
Very sparse	0.96 [0.73; 1.24]	0.734	1.51 [0.93; 2.44]	0.098
* A private space available in the accommodation if the child needed it*				
No (Ref)	-		-	
**Yes**	**0.49 [0.38; 0.64]**	**< 0.001**	**0.26 [0.17; 0.38]**	**< 0.001**
**Child mental health-related behaviors**				
*How frequently the child went outside during the lockdown*				
**Never**	**1.65 [1.15; 2.37]**	**0.006**	1.53 [0.78; 3.01]	0.215
**Approximately once a week**	**1.48 [1.08; 2.03]**	**0.015**	1.40 [0.75; 2.61]	0.293
Approximately 3 times a week	1.37 [0.99; 1.90]	0.057	0.86 [0.43; 1.71]	0.665
**Every day, once a day**	**1.42 [1.05; 1.92]**	**0.025**	1.27 [0.68; 2.38]	0.454
Multiple times a day (Ref)	-		-	
*Time spent every day on social media since the beginning of the lockdown*				
None (Ref)	-		-	
Less than an hour	0.71 [0.47; 1.07]	0.101	2.16 [0.81; 5.75]	0.124
1 to 3 h	1.01 [0.70; 1.47]	0.939	2.40 [0.94; 6.08]	0.066
3 to 5 h	1.17 [0.79; 1.72]	0.435	2.54 [0.98; 6.56]	0.055
**More than 5 h**	1.41 [0.94; 2.12]	0.098	**3.58 [1.38; 9.32]**	**0.009**
*Frequency of leisure and recreational activities with adults in the household since the beginning of the lockdown*				
**Never**	**1.53 [1.14; 2.04]**	**0.004**	**2.71 [1.60; 4.59]**	**< 0.001**
Approximately once a week	1.23 [0.95; 1.61]	0.119	1.40 [0.82; 2.37]	0.217
Approximately 3 times a week	1.10 [0.84; 1.45]	0.476	0.61 [0.33; 1.13]	0.117
Every day (Ref)	-		-	
*Time spent on school homework every day since the beginning of the lockdown*				
**Less than one hour**	**1.42 [1.08; 1.88]**	**0.012**	**1.97 [1.24; 3.12]**	**0.004**
One hour	1.10 [0.83; 1.44]	0.510	0.85 [0.50; 1.45]	0.555
2 to 3 h	1.12 [0.89; 1.42]	0.321	0.95 [0.60; 1.50]	0.827
More than 3 h (Ref)	-		-	
**Impact of the COVID-19 pandemic on relatives**				
*COVID-19-related infection and/or hospitalization of a loved one*				
No (Ref)	-		-	
Yes	1.04 [0.86; 1.26]	0.697	1.21 [0.85; 1.71]	0.286

aOR: adjusted Odds Ratios; 95% CI: 95% Confidence Interval; p: p-value; Ref: Reference

The results were adjusted for parent’s nationality, parent’s level of education, type of housing, history of mental health disorder and experience of a painful and/or stressful event in the previous 12 months

#### Protective factors for moderate psychological distress

The only protective factor for moderate psychological distress in children highlighted by multinomial logistic regression was the possibility of a private space within the living accommodation when needed (aOR, 0.49; 95% CI, 0.38; 0.64).

#### Risk factors for severe psychological distress

The risk factors most strongly associated with severe psychological distress (Table 4) in children were: parent’s occupation was a frontline worker (aOR, 1.66 ; 95% CI, 1.00 ; 2.75), spending more than 5 hours on social media a day since the lockdown started (aOR, 3.58 ; 95% CI, 1.38 ; 9.32), never doing any leisure and recreational activity with adults in the household since the lockdown started (aOR, 2.71 ; 95% CI, 1.60 ; 4.59), and doing less than one hour a day of school homework since the beginning of the lockdown (aOR, 1.97 ; 95% CI, 1.24 ; 3.12).

#### Protective factors for severe distress

The only protective factor for severe psychological distress highlighted by the multinomial logistic regression was the possibility of a private space within the living accommodation when needed (aOR, 0.26; 95% CI, 0.17; 0.38).

## Discussion

The results of the present study highlight the impact of structural social determinants, such as parents’ socio-demographic characteristics, housing and living conditions, on children’s psychological distress during the period of the first national lockdown. With regard to children’s socio-demographic characteristics, being a female was associated with moderate psychological distress. Concerning parent socio-demographic characteristics, the parent’s occupation - in particular being an essential frontline worker - was associated with severe psychological distress. In terms of living and housing conditions, parental financial difficulties were associated with moderate psychological distress. Furthermore, the possibility for the children to have a private space within the living accommodation when needed was a protective factor both for moderate and severe psychological distress. In terms of health behaviors, children with moderate distress in our study engaged less in physical activity (e.g., going outside); furthermore, the lack of leisure and recreational activities with adults in the household during the lockdown was a risk factor for both moderate and psychological distress. Doing less than one hour a day school homework since the beginning of the lockdown was associated with both moderate and severe psychological distress, while spending more than 5 hours a day on social media was associated with severe psychological distress.

In our study, as expected, being a female was associated with moderate psychological distress; this reflects findings from previous studies conducted in China [20], or in Brazil [21]. Even though females are usually more inclined to express their emotions, we hypothesize that the gendered distribution of domestic and educational tasks during the lockdown impacted females more, and that gender inequalities in terms of performing domestic tasks were accentuated [22, 23]. The national SAPRIS Elfe/Epipage2 survey, conducted in France in April-May 2020, found that girls were more involved in domestic chores in order to help parents cope with the increased pandemic-related domestic workload.

The results of our study show that children from socioeconomically disadvantaged families were at a greater risk of severe psychological distress. In univariable analysis, adolescents living with a single parent experienced more psychological distress than others (reflecting previous studies in France [25]). This factor was no longer significant in our multivariable model when financial difficulties were taken into account. The association between a parent being an essential frontline worker who had financial difficulties and psychological distress in the child confirms previous research indicating that financial hardship during the COVID-19 pandemic resulted in elevated stress levels while social inequalities strongly influenced the ways in which people experienced lockdowns [17, 23–33]. Similar associations between mental health and socio-economic factors during lockdowns have been found in other countries, such as in the UK [34] and in Canada [35].

Low-income households (which for the most part are immigrant households) living in poor housing conditions are overrepresented in the population of essential frontline workers in France [36]. One of the French government’s key measures during the pandemic was to prioritize the return to school of frontline healthcare workers’ children, but not necessarily of other frontline workers. This may explain why unlike many studies which highlighted a negative influence of the COVID-19 pandemic on the mental health of frontline healthcare workers [37–40], having a parent who was a healthcare professional was not associated with children psychological distress in our multivariable model.

Our results regarding the association between the health behaviors of children and their psychological distress are consistent with those from a similar study in France [18]. That study found that living conditions were associated with a low health-related quality of life among children during the COVID-19 lockdown. Increased screen time became an integral element of adapting to the COVID-19 pandemic. A large study of children and adolescents also linked smartphone addiction and excessive internet use to increased depression [41].

In our study, not having an appropriate place to self-isolate at home was related both to moderate and severe psychological distress. This result is congruent with that of Gatell-Carbó et al. who showed that children and adolescents with abnormal SDQ (Strengths and Difficulties Questionnaire) scores had substantially less personal space to study than the rest of their sample [43].

France’s first lockdown significantly disrupted school routines. Some parents, especially single parents, had to juggle between the huge burden of childcare, household chores, and teleworking which may have negatively impacted the time they could dedicate to homeschooling. Studies have highlighted that children from low-income families were less likely to have access to digital tools and a good quality internet connection to do their homework; they were also less likely to have an isolated room to work in during the lockdown [42, 43]. Consequently, distance learning during the pandemic aggravated educational inequalities in France and elsewhere [44, 45].

Although infection and/or hospitalization of a relative due to COVID-19 was not a factor for psychological distress in our multivariable model, its impact should not be ignored, as many studies have shown that it can affect the mental health status of young people [31, 41, 46–48]. In univariable analysis, infection and/or hospitalization of a relative due to COVID-19 was associated both with moderate and severe psychological distress, but was no longer significant in our multivariable model when being an essential frontline worker was taken into account. Indeed, previous studies have shown that COVID-19 related morbidity and mortality was higher in migrants [49], overrepresented in frontline workers [36].

### Strengths and limitations

The study has several limitations. First, a part of the data collection occurred during the two-month summer school holiday period (July-August 2020) in France when children would not normally have been at school. Ideally, data collection should have been concluded by the end of June. Second, school closure experiences were reported retrospectively; we had no data on when exactly the child returned to school or for how many hours per week. This made it impossible to directly measure the influence of returning to school on children mental health. Third, CONDEADO did not collect data on parents’ mental health. These could have been useful, especially for mothers, as they may have a greater influence on the emotional well-being of their children. Stress and anxiety levels related to the health crisis may have affected children’s mental health. Fourth, we chose to include children whose parents did not provide information about their occupation (the ‘unknown’ occupation category, see above). The presence of this ‘unknown’ modality in this variable may have led to potential bias in our results. Fifth, CONFEADO was conducted without a sample design and it is probable that families interested in mental health were more inclined to agree to participate. Nevertheless, considering a diverse range of social situations - through the collection of socio-demographic and environmental data from parents - allowed us to take into account social health inequalities.

Thus, our study has also several strengths. It is the first national study to assess the mental health and experiences of children during and after the first lockdown in France using self-evaluation. Furthermore, the CAPDS-10 scale has good psychometric characteristics and provides a reliable measure of distress at a given time [19]. Given its cross-sectional design, this study acknowledges the limitations in drawing causal relationships, but it still offers valuable evidence and sheds light on potential links between social determinants such as socio-economic circumstances and children’s psychological distress, during the pandemic contributing to our understanding of their mental health during this unprecedented crisis.

## Conclusions

Our results highlight the importance of having socially differentiated public policies, in particular to support and accompany families in fragile socioeconomic conditions in order that the needs of all children can be met. This was especially true for low-income frontline workers during the COVID-19 pandemic who had to continue to work outside of home while everyone else was expected to stay at home. In the event of future lockdowns (for whatever reason), educational continuity and the earliest possible return to the classroom are two actions which could help limit feelings of being overwhelmed by school work, and the related negative impact on the mental health of children. Protective factors such as the quality of relationships and screen-free activities within the family need to be reinforced through accessible and appropriate information for parents and children.

## Data Availability

The datasets generated and/or analyzed during the current study are not publicly available due to limitations associated with ethical approval. They are available from the corresponding author upon reasonable request.

## List of Abbreviations

aOR: Adjusted Odds Ratio
AP-HP: Assistance Publique-Hôpitaux de Paris
ASE: Aide Sociale à l’Enfance
CAPDS-10: Children and Adolescent’s Psychological Distress Scale
CBCL: Child Behavior Checklist
CI: Confidence Interval
CNIL: Commission nationale de l’informatique et des libertés
CONFEADO: CONFinement, Enfant et ADO
INSEE: Institut National de la Statistique et des Études Économiques
ONPE: Observatoire national de la protection de l’enfance
SDQ: Strengths and Difficulties Questionnaire
SpF: Santé publique France
YSR: Youth Self Report

## Footnotes

1 We performed a multivariable multinomial logistic regression model in an earlier version of the article before the DAG was developed following a reviewer’s suggestion (data not shown but available on request).
